# Assessing and Reassessing the Association of Comorbidities and Coinfections in COVID-19 Patients

**DOI:** 10.7759/cureus.36683

**Published:** 2023-03-25

**Authors:** Aryaan Khan, Ahmed El Hosseiny, Rania Siam

**Affiliations:** 1 Medicine, University of Medicine and Health Sciences, Basseterre, KNA; 2 Bioinformatics, The American University in Cairo, Cairo, EGY; 3 Microbiology and Molecular Sciences, The American University in Cairo, Cairo, EGY

**Keywords:** management of covid-19 patients, covid-19 infection, cancer and covid-19, covid-19 and asthma, covid-19 and diabetes milletus, hypertension and covid-19, coinfection in covid-19, covid-19, antimicrobial resistance in covid-19, covid-19 comorbidity

## Abstract

Coronavirus disease 2019 (COVID-19) has posed an enormous global health and economic burden. To date, 324 million confirmed cases and over 5.5 million deaths have been reported. Several studies have reported comorbidities and coinfections associated with complicated and serious COVID-19 infections. Data from retrospective, prospective, case series, and case reports from various geographical locations were assessed, which included ~ 2300 COVID-19 patients with varying comorbidities and coinfection. We report that Enterobacterales with *Staphylococcus aureus* was the most while *Mycoplasma pneumoniae* was the least prevalent coinfection in COVID-19 patients with a comorbidity. In this order, hypertension, diabetes, cardiovascular disease, and pulmonary disease were the prevalent comorbidities observed in COVID-19 patients. There was a statistically significant difference in the prevalent comorbidities observed in patients coinfected with S*taphylococcus aureus* and COVID-19 and a statistically non-significant difference in the prevalent comorbidities in patients coinfected with *Mycoplasma pneumoniae* and COVID-19 as compared to similar infections in non-COVID-19 coinfection. We report a significant difference in the prevalent comorbidities recorded in COVID-19 patients with varying coinfections and varying geographic study regions. Our study provides informative data on the prevalence of comorbidities and coinfections in COVID-19 patients to aid in evidence-based patient management and care.

## Introduction and background

Introduction

The management of severe coronavirus disease 2019 (COVID-19) is a global concern. More than 6.55 million people have died from severe COVID-19 infections. The severity of COVID-19 infection has multiple factors; however, comorbidities and coinfections are some that have significance. The management of COVID-19 differs depending on severity. As recommended by the WHO, severe cases of COVID-19 with antimicrobials as coinfections are prevalent. In the first six months of the pandemic, there was no significant difference in the mean antibiotic prescribing rate between patients with severe or critical illness versus patients with mild or moderate illness [1]. Antibiotic prescription for mild and moderate COVID-19 patients worldwide was inconsistent with WHO and UK NICE (National Institute for Health and Care Excellence) guidelines [1]. There has been widespread use of antibiotics in the treatment of COVID-19 regardless of secondary bacterial infection [1]. Yet, coinfections increase the severity of COVID-19 cases [2]. COVID-19 patients with comorbidities increase their risk of developing coinfections [2]. Understanding the relationship between coinfections and comorbidities in different patient populations will aid physicians in COVID-19 patient management. Evidence-based COVID-19 guidelines are a necessity for developing antimicrobial stewardship.

Previous reports showed that diabetes was the most prevalent comorbidity observed in COVID-19 patients coinfected with Mucormycetes [3]. Pulmonary disease was the most prevalent comorbidity observed in COVID-19 patients coinfected with *Pseudomonas aeruginosa* [4] while hypertension was the most prevalent comorbidity recorded in COVID-19 patients coinfected with *Staphylococcus aureus* [5]. Few studies discuss the secondary pathogen in COVID-19 coinfections and the patients' associated comorbidities observed. Our study compiles data from associated coinfections with specific comorbidities in COVID-19 patients and discusses the different prevalent comorbidities and coinfections in COVID-19 patients as compared to non-COVID-19 patients.

Methods

COVID-19 patient data were compiled from retrospective and prospective studies, case series, and case studies from various geographic locations during the COVID-19 pandemic. Comorbidities from over 2300 COVID-19 patients who had a coinfection additional to their COVID-19 infection were analyzed from 58 different studies [3-60]. Comorbidities from over 145,000 patients without COVID-19 were analyzed from 10 different studies [61-70]. Each study was about a different pathogen. Coinfections, comorbidities, and the region in which the study was conducted were analyzed and compiled. The 11 most prevalent coinfection-causing pathogens in COVID-19 patients were examined. Comorbidities were subcategorized into their respective groups (Table 1).

**Table 1:** Comorbidity classification

Categorized Comorbidity	Patient Comorbidity
Cardiovascular Disease	Coronary Artery Disease, Myocardial Infarction, Heart Failure, Cardiomyopathy, Stroke, Deep Vein Thrombosis, Peripheral Artery Disease, Aortic Disease, Ischemic Heart Disease, Cerebrovascular Disease, Pulmonary Vascular Disease
Cancer	Atrial Myxoma, Breast Cancer, Cancer, Malignancy, B-Cell Leukemia
Neurological Disease	Neurological Condition, Dementia
Renal Disease	Chronic Kidney Disease, Renal Disease, Chronic Renal Failure, Kidney Disease, Hemodialysis
Liver Disease	Chronic Liver Disease, Hepatitis B Virus, Hepatitis C Virus, Liver Cirrhosis, Liver Disease
Pulmonary Disease	Asthma, Chronic Obstructive Pulmonary Disease, Lung Condition, Chronic Lung Disease, Pulmonary Fibrosis, Respiratory Disease
Hypertension	Arterial Hypertension

A total of 70 studies were identified from Google Scholar, of which five were excluded due to overlapping data. Six additional studies were excluded due to missing comorbidity patient data, leaving a total of 57 studies included in the review.

A Pearson correlation analysis was conducted on the relationship between comorbidities, secondary, and coinfection in COVID-19 patients. A chi-squared analysis was conducted as well as a paired T-test when comparing the prevalent coinfections reported in COVID-19 cases with comorbidities versus non-COVID-19 cases with the same underlying medical condition.

## Review

Results

Pathogens Contributing to the Coinfections in COVID-19 Patients With Comorbidities

HIV, Candida, *Staphylococcus aureus*, *Pseudomonas aeruginosa*, *Mycoplasma pneumoniae*, Chlamydia, Mucormycetes, influenza, and Enterobacterales were reported coinfections in COVID-19 patients (Table 2). Additionally, *Mycoplasma pneumoniae* with Chlamydia and Enterobacterales with *Staphylococcus aureus* were reported in selected COVID-19 patients (Table 2). Enterobacterales with *Staphylococcus aureus* were the most prevalent coinfections in COVID-19 patients with a comorbidity; this was irrespective of the type of comorbidity (Table 2). *Mycoplasma pneumoniae* was the least prevalent coinfection in COVID-19 patients with a comorbidity (Table 2).

**Table 2:** Total number of COVID-19 cases in patients with a comorbidity and coinfected by a pathogen

Type of Pathogen	# of COVID-19 Coinfections
HIV	172
Candida	155
Staphylococcus aureus	160
Pseudomonas aeruginosa	235
Mycoplasma pneumoniae & Chlamydia	249
Mycoplasma pneumoniae	115
Mucormycetes	218
Influenza	272
Enterobacterales	118
Enterobacterales & Staphylococcus aureus	359
Aspergillus	252

**Figure 1:**
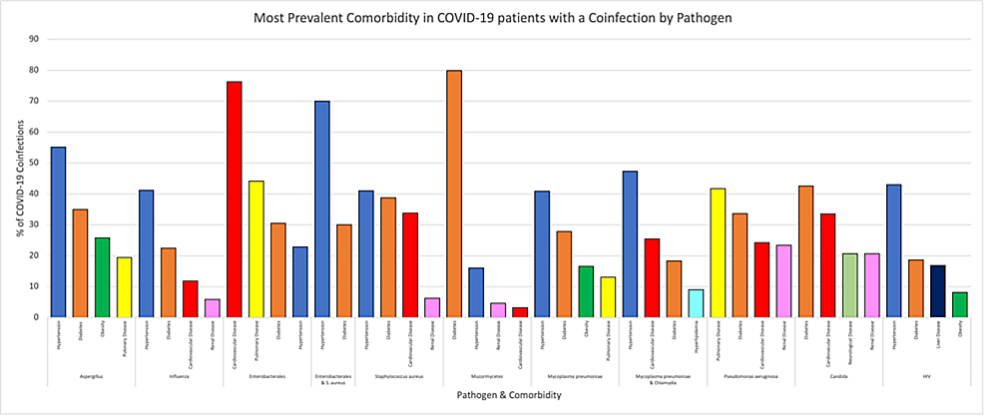
Prevalent comorbidities observed in COVID-19 patients with a coinfection, categorized by pathogen A chi-squared analysis was conducted to determine statistical significance (p<0.05). There was a statistically significant difference in the prevalent comorbidities observed between all groups of pathogens.

There was a significant difference in the most common comorbidities in patients coinfected with *Staphylococcus aureus* and COVID-19 compared to non-COVID-19 coinfected with *Staphylococcus aureus* (Table 3). There was a statistically non-significant difference in the most common comorbidities in patients coinfected with *Mycoplasma pneumoniae* and COVID-19 compared to non-COVID-19 coinfected with *Mycoplasma pneumoniae* (Table 3). The difference in COVID-19 comorbidities in patients coinfected with Aspergillus, influenza, Enterobacterales, Mucormycetes, *Pseudomonas aeruginosa*, Candida, and HIV could not be statistically evaluated due to the lack of sufficient data for statistical analysis (Table 3). Yet, there's an apparent trend difference in comorbidities observed in COVID-19 patients coinfected with the former pathogens (Figure 1).

**Table 3:** Prevalent comorbidities reported in specific infections with a COVID-19 coinfection compared to specific infections without a COVID-19 coinfection (++) indicates there is a significant difference between groups coinfected with COVID-19 and without. (--) indicates there was statistical insignificance. CVD: cardiovascular disease

Pathogen	Comorbidity	With COVID-19 (%)	Without COVID-19 (%)
*Staph. aureus* ++	Hypertension	41	N/A
	Diabetes	38.75	22.08
	CVD	33.75	17.70
	Neurological Disease	N/A	17.63
	Neoplastic Disease	3.75	15.44
	Pulmonary Disease	5.00	13.40
	Renal Disease	6.25	12.28
	Liver Disease	0.63	6.97
Aspergillus	Hypertension	55.16	N/A
	Diabetes	34.92	18.97
	Obesity	25.79	N/A
	CVD	19.44	23.28
	Pulmonary Disease	18.25	60.34
	Autoimmune Disease	N/A	5.17
Influenza	Hypertension	41.18	51.37
	Diabetes	22.43	20.39
	CVD	11.76	41.18
	Renal Disease	5.88	18.82
	Chronic Respiratory Disease	4.40	27.06
	Liver Disease	2.20	7.06
Enterobacterales	Cancer	4.24	56.57
	CVD	76.27	35.43
	Pulmonary Disease	44.07	2.86
	Diabetes	30.51	22.86
	Hypertension	22.88	N/A
	Renal Disease	4.24	14.29
	Liver Disease	1.70	9.71
	Ulcerative Disease	N/A	6.29
	Collagen Disease	N/A	4
	Neurological Disease	13.56	3.43
Mucormycetes	Diabetes	79.82	36
	Hypertension	16.06	N/A
	Cancer	2.30	28
	Renal Disease	4.59	N/A
	CVD	3.21	N/A
	HIV	N/A	2
	Autoimmune condition	N/A	1
*Mycoplasma pneumoniae* --	Hypertension	40.87	4.16
	Diabetes	27.83	2.43
	Obesity	16.52	N/A
	Pulmonary disease	13.04	19.30
	Hyperlipidemia	2.60	1.23
	CVD	7.82	1.99
	Liver Disease	1.74	0.95
	Cancer	0.87	0.74
Pseudomonas aeruginosa	Pulmonary disease	41.70	N/A
	CVD	24.26	37.70
	Diabetes	33.62	25.10
	Hematological disease	N/A	23.30
	Solid organ cancer	N/A	18.10
	Chronic lung disease	N/A	16.30
	Renal Disease	23.40	10.20
	Liver disease	N/A	13.50
Candida	Diabetes	42.58	21.50
	Cardiovascular Disease	33.55	N/A
	Neurological Disease	20.65	N/A
	Renal Disease	20.65	N/A
	Cancer	6.45	21.50
	AIDS	N/A	15.40
	Respiratory disease	16.77	14.00
	Transplant	N/A	4.60
HIV	Hepatitis	N/A	51.60
	Hypertension	43.02	4.9
	Mental health	N/A	22.50
	CVD	N/A	20.40
	Diabetes	18.60	11.20
	Liver disease	16.86	5.60
	Obesity	8.14	N/A

Prevalent Comorbidities in COVID-19 Patients With Specific Coinfections

**Figure 2:**
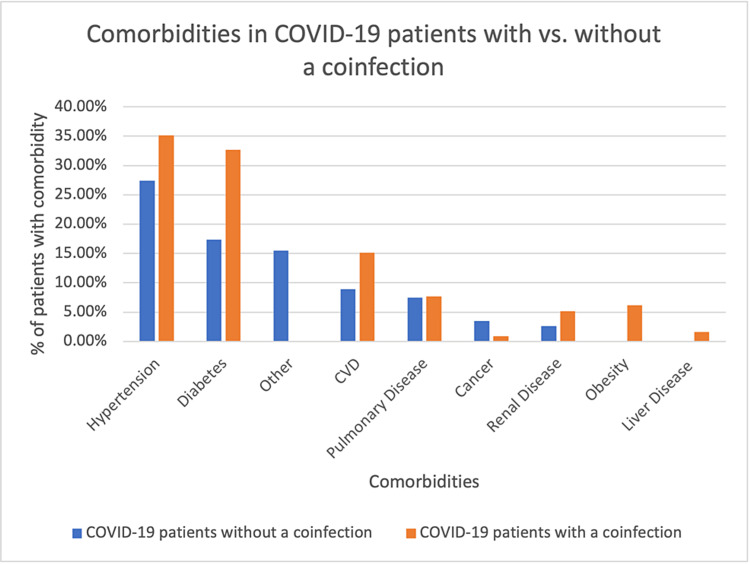
Prevalence of comorbidities in COVID-19 cases with and without a coinfection A chi-squared analysis was conducted to determine statistical significance (p<0.05). There was a statistically significant difference between the group of COVID-19 patients without a coinfection compared to those with a coinfection.

Hypertension, diabetes, cardiovascular, pulmonary and renal diseases, obesity, cancer, and liver diseases were the prevalent comorbidities reported in COVID-19 patients (Table 4). In this order, hypertension, diabetes, cardiovascular disease, and pulmonary disease were the most prevalent comorbidities observed in COVID-19 patients regardless of the pathogen (Table 4). There was a significant difference in prevalent comorbidities observed in COVID-19 patients with a coinfection compared to COVID-19 patients without a coinfection (Figure 2).

**Table 4:** Prevalent comorbidities recorded in COVID-19 patients with a coinfection, regardless of the pathogen

Type of Comorbidity	# of COVID-19 Coinfections
Hypertension	807
Diabetes	754
Cardiovascular Disease	423
Pulmonary Disease	273
Renal Disease	179
Obesity	127
Cancer	93
Liver Disease	86

Hypertension was the most prevalent comorbidity in COVID-19 patients coinfected with Aspergillus, influenza, Enterobacterales, *Staphylococcus aureus*, *Mycoplasma pneumoniae*, Chlamydia, and HIV (Figure 2). Diabetes was the most prevalent comorbidity in COVID-19 patients coinfected with Mucormycetes and Candida. Pulmonary disease was the most prevalent comorbidity in COVID-19 patients coinfected with *Pseudomonas aeruginosa*. Cardiovascular disease was the most prevalent comorbidity in COVID-19 patients coinfected with Enterobacterales (Figure 2). The prevalent comorbidities reported in one pathogen, illustrated in Figure 2, are all significantly different from the prevalent comorbidities reported in the other pathogens.

**Figure 3:**
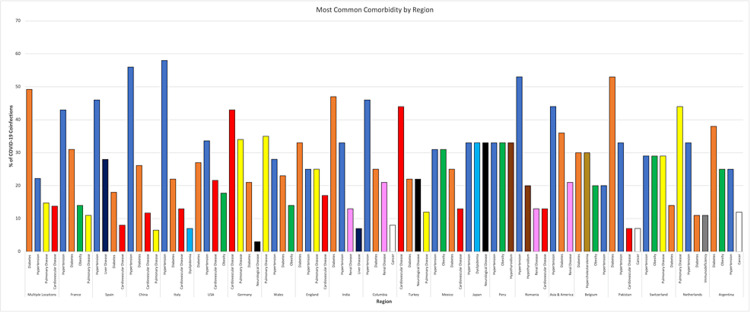
Regional categorization of prevalent comorbidities recorded in COVID-19 patients with a coinfection A chi-squared analysis was conducted to determine statistical significance (p<0.05). There was a statistically significant difference in the prevalent comorbidities recorded between every region.

Hypertension was the most prevalent comorbidity in coinfected COVID-19 patients in studies conducted in France, Spain, China, Italy, Columbia, Mexico, Peru, Romania, Asia, America, and Switzerland (Figure 3). Diabetes was the most prevalent comorbidity in coinfected COVID-19 patients in studies conducted in multiple countries, including India, the USA, England, Belgium, Pakistan, and Argentina. Cardiovascular disease was the most prevalent comorbidity in coinfected COVID-19 patients in studies conducted in Germany and Turkey. Pulmonary disease was the most prevalent comorbidity in coinfected COVID-19 patients in studies from Wales and the Netherlands (Figure 3). The prevalent comorbidities reported in one region, illustrated in Figure 3, are all significantly different from the prevalent comorbidities reported in the other regions. Our data evaluates the prevalent comorbidities and pathogens contributing to the coinfections in COVID-19 patients.

Influenza was the most prevalent coinfection seen in a COVID-19 patient with a comorbidity in China, whereas *Mycoplasma pneumoniae* and Chlamydia was the most prevalent coinfection seen in a COVID-19 patient with a comorbidity in Italy (Table 5).

**Table 5:** Regional categorization of prevalent coinfections recorded in COVID-19 patients with a comorbidity

Location of Study	Type of Pathogen	# of COVID-19 Coinfections
Multi-national	Mucormycosis	214
	Pseudomonas aeruginosa	205
	Staphylococcus aureus	115
	Candida	43
	HIV	43
	Aspergillus	38
	Influenza	37
France	Aspergillus	76
Spain	HIV	51
	Enterobacterales	30
	Aspergillus	7
China	Influenza	188
	Mycoplasma pneumoniae	22
Italy	*Mycoplasma pneumoniae* & Chlamydia	249
	HIV	47
	Aspergillus	30
	Enterobacteriaceae	7
	Staphylococcus aureus	1
	Candida	1
USA	Candida	128
	Mycoplasma pneumoniae	85
	Staphylococcus aureus	42
	Aspergillus	39
	HIV	31
	Influenza	8
Germany	Enterobacteriaceae	67
	Pseudomonas aeroginosa	22
	Aspergillus	5
Wales	Aspergillus	25
	Candida	14
England	Mycoplasma pneumoniae	8
	Staphylococcus aureus	2
India	Candida	15
	Mucormycetes	10
Colombia	Candida	39
Turkey	Candida	32
Mexico	Pseudomonas aeroginosa	8
Japan	Enterobacteriaceae	1
Peru	Enterobacteriaceae	4
Romania	Enterobacteriaceae	9
Asia & America	Influenza	29
Belgium	Aspergillus	6
Pakistan	Aspergillus	9
Switzerland	Aspergillus	3
Netherlands	Aspergillus	9
Argentina	Aspergillus	5

Discussion

As demonstrated by previous literature, COVID-19 patients with comorbidities have an increased risk of developing coinfections, which also increases the severity of COVID-19 cases [2]. As recommended by the WHO, severe cases of COVID-19 require the use of antimicrobials as prophylactic treatment [1]. This study aims to provide meaningful data to determine which prophylactic treatment is best suited for a specific patient population.

Considering *Pseudomonas aeruginosa* is the most prevalent confection in COVID-19 patients with pulmonary disease, COVID-19 patients with pulmonary disease should be closely monitored for signs and symptoms of *Pseudomonas aeruginosa* infections. Prophylactic treatment against *Pseudomonas aeruginosa* and its benefits should be investigated in COVID-19 patients with a pulmonary disease comorbidity. As a consequence of Enterobacterales being the most prevalent coinfection in COVID-19 patients with cardiovascular disease (CVD), COVID-19 patients with CVD should be closely monitored for signs and symptoms of Enterobacterales infections. Prophylactic treatment against Enterobacterales should be considered by physicians, and its benefits should be investigated in COVID-19 patients with a CVD comorbidity. Mucormycosis is the most prevalent infection reported in diabetic patients [3]. This remains to be true in COVID-19 patients as well. Due to Mucormycosis being the most prevalent coinfection in COVID-19 patients with diabetes, COVID-19 patients with diabetes should be closely monitored for signs and symptoms of Mucormycosis infections. Physicians should consider prophylactic treatment against Mucormycosis and its benefits should be investigated in COVID-19 patients with a diabetes comorbidity.

The results of this study also highlighted several important conclusions on COVID-19 precautions in various countries. Due to influenza being the most prevalent coinfection in COVID-19 patients with a comorbidity in China, COVID-19 patients with a comorbidity in China should be closely monitored for signs and symptoms of influenza infections. Additionally, considering *Mycoplasma pneumoniae* and Chlamydia is the most prevalent coinfection in COVID-19 patients with a comorbidity in Italy, COVID-19 patients with a comorbidity in Italy should be closely monitored for signs and symptoms of *Mycoplasma pneumoniae* & Chlamydia infections. Prophylactic treatment against *Mycoplasma pneumoniae* and Chlamydia should be considered by physicians, and its benefits should be investigated in COVID-19 patients with a comorbidity in Italy.

Other studies have illustrated which comorbidities are more prevalent in specific coinfections. For example, Pal et al. highlighted that diabetes was the most prevalent comorbidity in patients coinfected with Mucormycetes and COVID-19 [3]. Another study found hypertension to be the most prevalent comorbidity in patients coinfected with* S. aureus* and COVID-19 [5]. However, there are no studies that compile relevant and up-to-date data regarding coinfections and comorbidities in COVID-19 patients into a literature review. Our study has bridged the gap between different studies and compiled data from the literature regarding coinfections and comorbidities in COVID-19 patients. This study's limitation is the number of research articles published on the prevalence of comorbidities in COVID-19 patients with a specific coinfection. Our study comprehensively only investigates all published and relevant literature. Future epidemiological studies will enhance our understanding of the relationship between COVID-19 coinfections and comorbidities and will provide a platform to aid physicians in COVID-19 patient management. Another limitation of this study is the global distribution of the research articles that were compiled. As noted in Table 5, a majority of the research articles were from countries within Asia, Europe, and North America. Although there are studies from other countries as well, future steps for this study would entail including a more balanced representation of regions within the results. This would enhance the translatability of the results, and strengthen the conclusions. Lastly, an additional limitation of this study is the nature of severe COVID-19 and the documentation of such cases. Severe COVID-19 can be rapidly deteriorating and, as a result, many hospitals may not have always documented coinfected cases that resulted in death. This may have affected the results within the research articles themselves and thus impacted the conclusions demonstrated in this study.

## Conclusions

In conclusion, this study demonstrates data regarding the prevalence of comorbidities and coinfections in severe COVID-19 patients. Future studies may enhance the data to include more pathogens and discuss other comorbidities. Evidence-based COVID-19 guidelines are a necessity for patient management and developing antimicrobial stewardship. Understanding the relationships between comorbidities and secondary coinfections in COVID-19 patient populations will aid physicians in evidence-based patient management and care.
